# Physicians’ Mutual Benefit Association

**Published:** 1889-07

**Authors:** 


### THE PHYSICIANS' MUTUAL BENEFIT ASSOCIATION OF TEXAS.

*Requiescat in Pace.*

After five years struggle to establish a benevolent feature in the medical profession of Texas, we have been compelled to give it up for want of proper co-operation and support on the part of the brethren. The membership has ranged in that time from fifty to one hundred and fifty-three, and the sums disbursed to the widows of deceased brethren, from fifty-five, to one-hundred and fifty-three dollars.

When assessments Nos. 9, 10 and 11 were issued, there were one hundred and twenty members. Of this number only sixty had paid at the expiration of the time fixed; and rather than close the account, and shut off delinquents, (in as much as some were absent, and some did not get the first notice) in obedience to instructions from the President, the time was extended, and second and third notices sent to them. As a result, seventy-nine dollars (net) was collected for No. 9 (Buck; receipt of Mr. Earle, for the amount, in Miss Buck's name, was published in the May number,) and seventy-three dollars each (net) for Nos. 10 and 11, (Maddox and Jameson) while for No. 12 (Ross) only fifty-nine dollars (net) was collected. The receipts of Mrs. Ross, Mrs. Maddox and Dr. Jameson, together with the name of every member who paid, will be published in the August number of the JOURNAL, together with a statement of disbursements in full from beginning to end; it was prepared for this number, but crowded out.

While our efforts to form a charitable feature in our ranks has not met the support it deserves, it is something to have collected some twelve or thirteen hundred dollars, and turned it over to the widows of our departed brethren; and to know that in so doing, real distress was relieved or prevented. To the few who have remained faithful to the end, we tender our grateful acknowledgments; and now surrender the effort with regret.—No more assessments will be issued.

